# Underlying mechanisms in the relationship between stress and alcohol consumption in regular and risky drinkers (MESA): methods and design of a randomized laboratory study

**DOI:** 10.1186/s40359-022-00942-1

**Published:** 2022-10-15

**Authors:** Charlotte Wittgens, Markus Muehlhan, Anja Kräplin, Max Wolff, Sebastian Trautmann

**Affiliations:** 1 Department of Psychology, Faculty of Human Science, Medical School Hamburg, Hamburg, Germany; 2 ICPP Institute for Clinical Psychology and Psychotherapy, Medical School Hamburg, Hamburg, Germany; 3 ICAN Institute for Cognitive and Affective Neuroscience, Medical School Hamburg, Hamburg, Germany; 4 Work Group Addictive Behaviors, Risk Analysis and Risk Management, Faculty of Psychology, Technische University Dresden, Dresden, Germany; 5 Department of Psychiatry and Psychotherapy, Campus Charité Mitte, Charité – Universitätsmedizin Berlin, corporate member of Freie Universität Berlin and Humboldt-Universität zu Berlin, Berlin, Germany

**Keywords:** AUD, Risky alcohol consumption, Acute stress, TSST, Ad-libitum taste-test

## Abstract

**Background:**

Excessive alcohol consumption and alcohol use disorders (AUD) are among the leading preventable causes of premature morbidity and mortality and are considered a major public health concern. In order to reduce the individual and societal burden of excessive alcohol use, it is crucial to identify high-risk individuals at earlier stages and to provide effective interventions to prevent further progression. Stressful experiences are important risk factors for excessive alcohol consumption and AUDs. However, the underlying biological and psychological mechanisms are still poorly understood.

**Methods:**

The project “Underlying mechanisms in the relationship between stress and alcohol consumption in regular and risky drinkers (MESA)” is a randomized controlled study that started in December 2018 and is conducted in a laboratory setting, which aims to identify moderators and mediators of the relationship between acute stress and alcohol consumption among regular and risky drinkers. Regular and risky drinkers are randomly assigned to a stress induction or a control condition. Several processes that may mediate (emotional distress, endocrine and autonomic stress reactivity, impulsivity, inhibitory control, motivational sensitization) or moderate (trait impulsivity, childhood maltreatment, basal HPA-axis activity) the relation between stress and alcohol consumption are investigated. As primary dependent variable, the motivation to consume alcohol following psychosocial stress is measured.

**Discussion:**

The results of this study could help to provide valuable targets for future research on tailored interventions to prevent stress-related alcohol consumption.

## Background

Excessive alcohol consumption and alcohol use disorders (AUD) are among the leading preventable causes of premature morbidity and mortality [1, 2]. They come along with an immense individual and societal burden and are considered a major public health problem [3]. The World Health Organization reported 3 million deaths due to harmful use of alcohol in their latest report [4]. In the age group 20–39 years, approximately 13.5% of the total deaths are attributable to alcohol [5]. In particular, men are considered at high risk to develop AUD [2] with global prevalence five times that in women with 8.6% and 1.7% for males and females, respectively [4]. However, latest data indicated that this gap is narrowing in recent years [6, 7]. Treatments for excessive alcohol use and AUD are initiated at a very late stage of symptom progression when adverse somatic and mental consequences have already occurred [8, 9]. It is therefore necessary to identify high-risk individuals at an earlier stage of alcohol consumption in order to reduce individual and societal burden and to implement effective interventions to prevent further progressions. Risk factors and underlying mechanisms promoting excessive alcohol use need to be identified for tailoring new preventive approaches.

### Stress and alcohol use

Alcohol consumption is a commonly used coping strategy to reduce stress [10]. It is very well known that Stress increases the amount of alcohol consumed and the risk of relapse, but little is known about the psychological mechanisms that underlie these effects [11]. The experience of stressful events, defined as unpredictable or uncontrollable events that exceed the regulatory capacity of an organism and that could threaten an organism’s physical or psychosocial integrity [10, 11] has been identified as a major risk factor for excessive alcohol use and AUD [12, 13]. The impact of stress on alcohol use and the risk of AUDs depends on the type, age, duration, and severity of the stress experienced [14]. The consumption of alcohol is a habitual response to stressful situations in people with AUD [15]. Stress plays an important role at all levels of alcohol consumption, beginning with facilitation of initial use through early stages of transition to regular use and from regular to excessive use [16, 17, 18]. In AUD, alcohol use also represents a habitual response to stressful situations [15].

### Mediators and moderators

Despite this well-established association between stress and alcohol use, the underlying mechanisms are complex and still not well understood. Studies trying to explain this association show inconsistent results. Stress does not necessarily lead to alcohol consumption in every person [19], which suggests the relevance of potential moderating factors. Several environmental, biological, and psychological factors that could moderate the relation between stress and alcohol consumption at different stages of alcohol use progression are discussed in the existing literature. The hypothalamic–pituitary–adrenal (HPA) axis plays an important role in this context as it is a major stress response pathway and has been studied extensively in relation to alcohol use [20]. Altered HPA axis regulation is associated with problematic alcohol use and dependence and the nature of this dysregulation varies with respect to the stages of progression toward AUD [21]. Glucocorticoid secretion upon activation of (HPA) axis by stressors is normally adaptive, and was discussed to promote coping after stressful events whereas excessive and prolonged HPA axis activation results in wear-and-tear on numerous physiological systems [22]. Furthermore, dysregulation in stress-related cortisol production is a risk factor for developing AUD [20]. Therefore, studies suggest that there might be a moderating effect on the relationship between stress and alcohol consumption by individual differences in basal cortisol secretion [18, 19]. Further, there is evidence from observational studies that childhood maltreatment moderates the association between stressful experiences and the development of alcohol use problems [23, 24]. Individuals with childhood trauma exposure, particularly abuse, neglect, or chaotic home environments, are at heightened risk for heavy alcohol consumption [24]. Further childhood maltreatment is associated with early alcohol use initiation, alcohol-related problem behaviors, and alcohol use disorders in adulthood [25]. Other possible moderators considered in this context are personality traits. Personality traits such as trait impulsivity reflect people’s characteristic patterns of thoughts, feelings, and behaviors and imply consistency over time and stability across situations [26]. Trait impulsivity was found to predict risk for alcohol use problems in general [27, 28, 29] and further moderates the association between stress and alcohol use [30, 31]. Although the consideration of these moderating factors might help to elucidate previous inconsistent findings on the association between stress and alcohol use and develop more targeted interventions, they have barely been considered in studies on its underlying mechanisms.

Regarding the underlying mechanisms of the relationship between stress and alcohol consumption, the idea of alcohol use as a dysfunctional coping strategy to self-medicate aversive emotional states following stressful experiences has long been the predominant model [32]. Although there is considerable empirical support for the self-medication hypothesis [32, 33, 34, 35], it is not able to fully explain the association between stressful experiences and alcohol use. Alcohol consumption does not necessarily reduce aversive emotional states [36, 37], violating the negative reinforcement assumption underlying the self-medication hypothesis. Therefore, knowledge on additional mechanisms beyond self-medication at different stages of alcohol use progression is required to explain the association between stress and alcohol use. Several relevant psychological and biological factors that might affect this relationship have been described in the literature [20, 38]. Acute stress activates an immediate reaction increasing cerebral and peripheral adrenalin and noradrenalin and a delayed endocrine response (via HPA axis) increasing glucocorticoids (mainly cortisol in humans) [39]. These systems affect different mechanisms relevant to alcohol use depending on the stage of alcohol use progression. At early stages of alcohol use progression, alcohol use leads to increased autonomic arousal and HPA axis activation. These effects potentiate both stress and alcohol-related effects on motivation and reinforcement learning [40] which can further facilitate alcohol use as a stress-related coping mechanism [38]. It further promotes the salience of drug-related cues known as attentional bias as these cues ‘grab the attention’ and further increase alcohol craving [41]. At later stages of alcohol use progression, binge and excessive alcohol consumption results in larger-scale adaptations in terms of a neuroendocrine tolerance response to stress and alcohol intake [10, 42] which may be involved in the transition from controlled to compulsive alcohol consumption [10, 43]. Also, a sensitization of motivational systems can manifest, again, in priority processing alcohol-related cues, i.e. attentional bias [44, 45]. The stress-induced sensitization at later stages of alcohol use progression is assumed to be active in parallel to the noradrenalin-related mechanisms [18]. Taken together, stress and stress system alterations by alcohol consumption could be associated with biased information processing, increased impulsivity and impaired control functions; a pattern that is known to be a key mechanism in the development of excessive alcohol use [46, 47].

### Need for controlled laboratory studies

Most studies, addressing the association between stress and alcohol consumption are based on clinical populations with limited sample sizes and participants who already developed AUD. In this context different moderators and mediators leading to alcohol dependence are often center of the research question [48, 49]. There is need for research that investigates the underlying mechanisms that lead to AUD before it is manifested. Therefore, especially laboratory settings with non-clinic samples are suitable to investigate mediators and moderators on this relationship as they allow the investigation of specific mechanisms through randomized manipulation of the factor of interest and at the same time allow to control for confounding variables [50].

### Aims and hypotheses

The present and ongoing study aims to fill this research gap by conducting an experimental laboratory design to investigate the underlying mechanisms of the association between stress and alcohol consumption (MESA) in the at-risk population of young men. Since these mechanisms are expected to differ depending on the stage of alcohol use, they are examined in regular and risky drinkers. Therefore, several processes that could mediate the relation between stress and alcohol consumption at different stages of alcohol use progression are assessed.

The research questions are as follows:

1. Does acute stress increase alcohol consumption in a laboratory setting?
2. What are the mediators of the association between acute stress and alcohol use?
3. What are the moderators of the association between acute stress and alcohol use?
4. Are effects of acute stress on alcohol use as well as moderators and mediators of this association different in risky drinkers compared to regular drinkers?

The following a priori hypothesis were formulated:

1. Acute stress increases alcohol consumption in a laboratory setting.
2. This effect is stronger in risky compared to regular drinkers.
3. Emotional distress, endocrine and autonomic stress reactivity as well as impulsivity account for most of the effect of stress on alcohol use in regular drinkers (mediation).
4. Emotional distress, endocrine and autonomic stress reactivity, impulsivity, attentional bias and craving account for most of the effect of stress on alcohol use in risky drinkers (mediation).
5. A history of childhood maltreatment, basal HPA-axis activity and impulsivity are related to a stronger effect of acute stress on alcohol consumption in regular and risky drinkers (moderation).

## Methods/design

### Study design

**Fig. 1:**
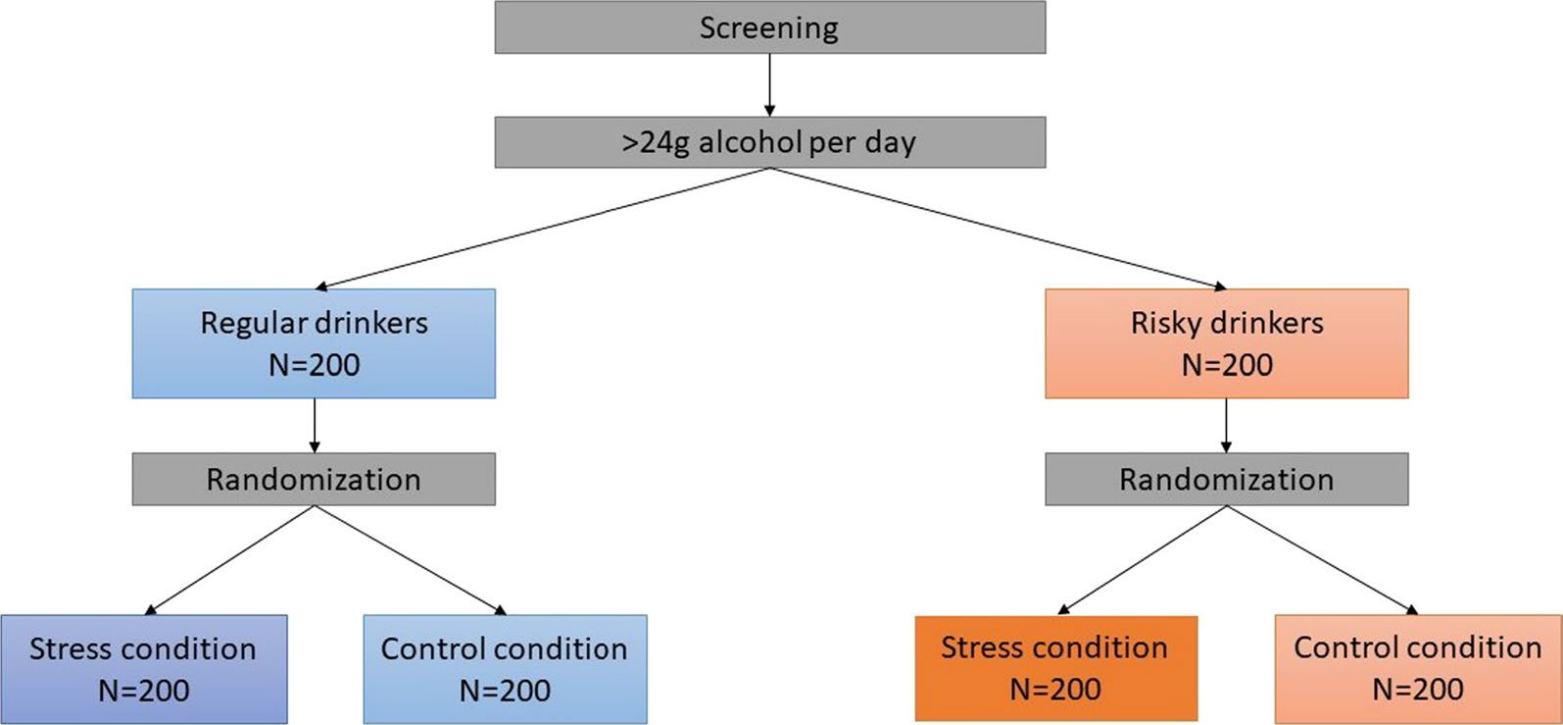
Study design

The MESA study is a randomized controlled study that started in December 2018 and is being conducted in a laboratory setting at the Medical School Hamburg. The study is divided into an online screening and a main examination, with detailed description in the following (“Procedure” section). The study has a four-group design. Participants are stratified into equal groups of regular and risky drinkers (with regular drinking being defined as average daily alcohol consumption of less than 24 g over the past 30 days and risky drinking being defined as average daily alcohol consumption of more than 24 g over the past 30 days [51]). Regular and risky drinkers are then randomly assigned to either an experimental (acute stress) or a control condition (Fig. 1).

Research is conducted in accordance with national data protection acts, the revised declaration of Helsinki and Good Clinical Practice Guidelines. After complete description of the study, written informed consent is obtained from all participants. The study is approved by the Institutional Review Boards of Technische Universität Dresden (EK 522122016) and Medical School Hamburg (MSH-2020/114).

### Inclusion and exclusion criteria

Males have a higher risk of developing drinking problems compared to females [52] and are more likely to report stress-induced drinking [6]. Therefore, only male individuals are included to reduce heterogeneity and potential of confounding factors (e.g. intake of oral contraceptives, menstrual cycle), especially in the biological measures. All participants have to be between 18–40 years old. The upper age limit results from the fact that most alcohol use problems develop during adolescence and young adulthood [53, 54, 55]. Further, eligible individuals have to drink alcohol at least occasionally and have beer as their favorite alcoholic drink since it is necessary for the success of the study that participants are familiar with alcoholic brands to recognize them in the attentional bias paradigm (“Assessment” section). In addition, it might be perceived as unethical to provide abstinent individuals with alcoholic beverages. Additionally, having a hair length of at least 2 cm is required to analyze hair cortisol concentrations [56] as a cumulative measure for basal cortisol secretion of the association between stress and alcohol use (for detailed description see 2.4 Biological measures). Exclusion criteria are lifetime psychotic symptoms, lifetime alcohol or any other substance use disorder, current psychological or psycho-pharmacological interventions and acute suicidality, current psychotropic or other medication or any somatic diseases that might confound the study measures, especially with regard to the endocrine measures, and alcohol consumption on the study day. All subjects meeting the inclusion criteria will be stratified into the reported groups.

### A priori power analysis

A non-clinical target sample of 400 young men is aimed for the MESA study. A power analysis was conducted to calculate the needed sample size. A series of Monte Carlo simulations (each simulating 1000 ANOVA F tests) using the simpower program in STATA 12.1 [57] was run. The Monte Carlo simulations revealed that assuming a sample size of n = 200 per drinking stratum, statistical power ranges between 0.80 and 0.95 for different group size ratios. Given the stratified randomized design of the study, this results in the final group size of n = 100 (Fig. 1).

### Recruitment and screening procedures

Participants are recruited via personal contacts, flyers and advertisement in university and public settings in Hamburg (cafés, bars, supermarkets, sports clubs; student dormitories) as well as via social media (e.g. Instagram, Facebook) and student job markets. In addition, advertising is made in lectures and on the university website.

All individuals willing to participate in the study have to complete an online screening in advance of the main assessment, where basic demographic variables as well as all in- and exclusion criteria are assessed. Further, the usual alcohol consumption is measured using a self-administered timeline follow-back consisting of a calendar on which participants provide retrospective reports of average daily alcohol intake for the past 30 days [58]. The information on daily alcohol consumption is used to allocate participants to the groups of regular and risky drinkers. All individuals meeting the inclusion criteria are then invited to participate in the main study.

### Assessment

#### Person-related measures

Participants complete a comprehensive baseline assessment (questionnaire package) including the measures of the proposed moderators (childhood maltreatment, trait impulsivity), mediators (attentional bias to alcohol related stimuli, inhibitory control and impulsivity, and stress reactivity during the acute stressor), and variables that might affect the associations of interest (usual alcohol consumption, drinking motives, perceived stress, trait anxiety, difficulties in emotion regulation, psychological flexibility) (Table 1).

**Table 1:** Variables and assessment instruments

Variable	Assessment	References
*Exclusion criteria*	Screening questions of the Structural Clinical Interview for DSM-IV	[59, 60]
Psychotic symptoms		
Suicidality		
Probable alcohol use disorder		
*Stratification variable*		
Usual alcohol consumption	Self-administered timeline followback	[58, 61]
*Moderators*		
Childhood adversities	Childhood trauma questionnaire (CTQ)	[62, 63]
Trait impulsivity	Impulsive Behavior Scale (UPPS)	[61, 64]
Cumulative cortisol secretion	Hair cortisol concentration	[56, 59]
*Mediators*		
Endocrine stress reactivity	Salvia cortisol concentration	[60, 65]
Autonomic stress reactivity	Salvia alpha amylase concentration	[63, 66]
Inhibitory control	Go-no-go task	[64, 67]
Attentional bias	Dotprobe task	[41]
Impulsivity	Delay discounting task	[68, 69, 70]
*Confounding variables*		
Current stress load	Perceived stress scale (PSS)	[67, 71]
Psychological flexibility	Acceptance and Action Questionnaire (AAQ-II)	[68, 72]
Drinking motives	Drinking Motive Questionnaire- revised (DMQ-R)	[69, 73]
Craving	Alcohol Craving Questionnaire revised (ACQ-R)	[71, 74]
Emotion regulation	Difficulties in Emotion Regulation Scale (DERS)	[75, 76]
Trait anxiety	State-Trait-Anxiety-Inventory (STAI)	[73, 77]
Positive and negative affect	State-Trait-Anxiety-Inventory (STAI)	[73, 77]
General condition	Multidimensional Mood State Questionnaire. (MBDF)	[74, 78]
Drug use	Drug screening	
Attentional control	Attentional control scale (ACS)	[75, 79]
Negative thinking	Perseverative thinking questionnaire (PTQ)	[77, 80]
Sleep quality	Pittsburgh sleep quality index (PTQI)	[78, 81]
Uncertainty intolerance	Uncertainty intolerance scale (UIS)	[79, 82]
Coping strategies	Brief Cope	[80, 83]
Depression	Becks Depression Inventory (BDI)	[81, 84]
Self-control	Brief self-control scale (BSCS)	[82, 85]
Resilience	Connor-Davidson Resilience Scale (CD-RISC)	[83, 86]
*Outcome*		
Momentary alcohol consumption	Ad-libitum taste test	[84, 87]

#### Biological measures

Hair strands are taken to reflect cumulative long-term cortisol secretion within two months prior to the respective assessment point [56]. The cumulative cortisol secretion consisting of basal cortisol secretion as well as stress-induced cortisol secretion, has been shown to be an important moderator of stress-related adverse consequences including increase in alcohol use [85, 86]. In addition, during the study four saliva samples are collected using Salivettes^®^ “code blue” (Sarstedt, Nümbrecht, Germany) with synthetic swabs to measure free cortisol levels and alpha-amylase activity as biological indicators of stress reactivity (Fig. 5). The first saliva sample is taken immediately before the stress induction (for detailed description for the stress induction see “Sample storage, biochemical analyses and data preparation” section). The second saliva sample is taken right after the stress induction as well as 12 (3rd salvia sample) and 24 (4th salvia sample) minutes after the stress induction. Cortisol is the final output of the hypothalamic pituitary adrenal (HPA) axis, and is among the most frequently used biological markers of psychological stress [87, 88]. Moreover, given that the biologically active (free) fraction of cortisol is reflected in saliva, it can be a preferred measure relative to serum cortisol [87, 89]. In addition, alpha-amylase is an enzyme component of saliva and has been proposed as a marker for stress-induced activity of the sympathetic nervous system (SNS). The advantage of a saliva-based measure of SNS activity is the convenience of assessing activity of both major stress systems (i.e. SNS and HPA-axis) in a single test tube, without the need for technically sophisticated instrumentation [63].

#### Behavioral measures

**Fig. 2:**
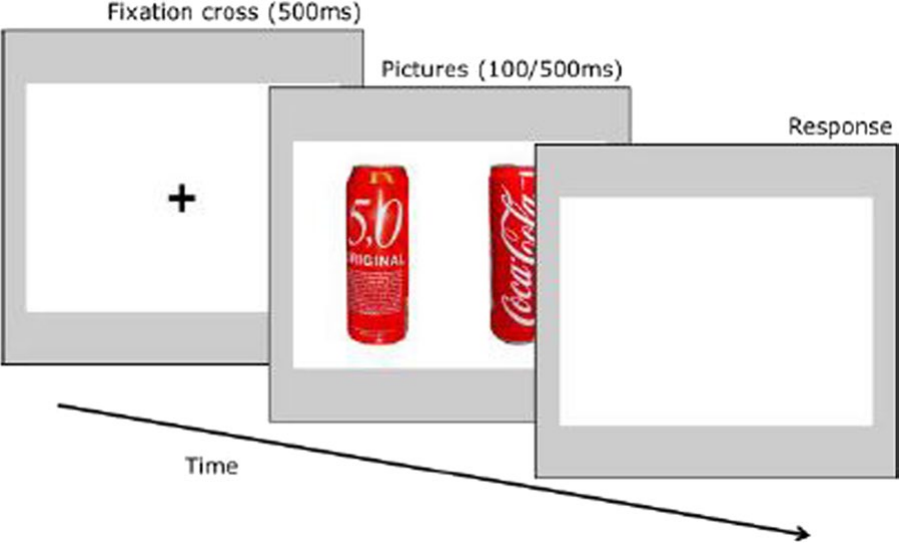
Dot-probe task

In addition to self-report measures, three behavioral tasks are conducted to measure attentional bias to alcohol related stimuli, inhibitory control and impulsivity as possible mediators in the association between stress and alcohol use [18, 90]. Attentional bias towards alcohol-related cues is measured using a dot-probe task (Fig. 2), which was programmed based on previous tasks in similar settings [41, 90]. Subjects are presented with pairs of matched alcoholic (beer) and non-alcoholic beverages for 500 ms (stimulus-onset asynchrony, SOA). Another SOA of 100 ms will be added to the paradigm in the proposed study to be able to capture automatic initial reactions (see [91]). Stimuli were chosen based on expert ratings regarding similarity in color, shape and recognition. Subjects respond to a probe that appears behind either the alcoholic or the non-alcoholic beverage. The difference in reaction time between alcoholic and non-alcoholic stimuli is a measure of attentional bias towards alcohol-related cues. Although the dot-probe task is a widely used paradigm to measure attentional biases, there is debate about its reliability [92, 93]. A new trial-based conceptualization of attentional bias has been proposed, which can increase reliability [94].

**Fig. 3:**
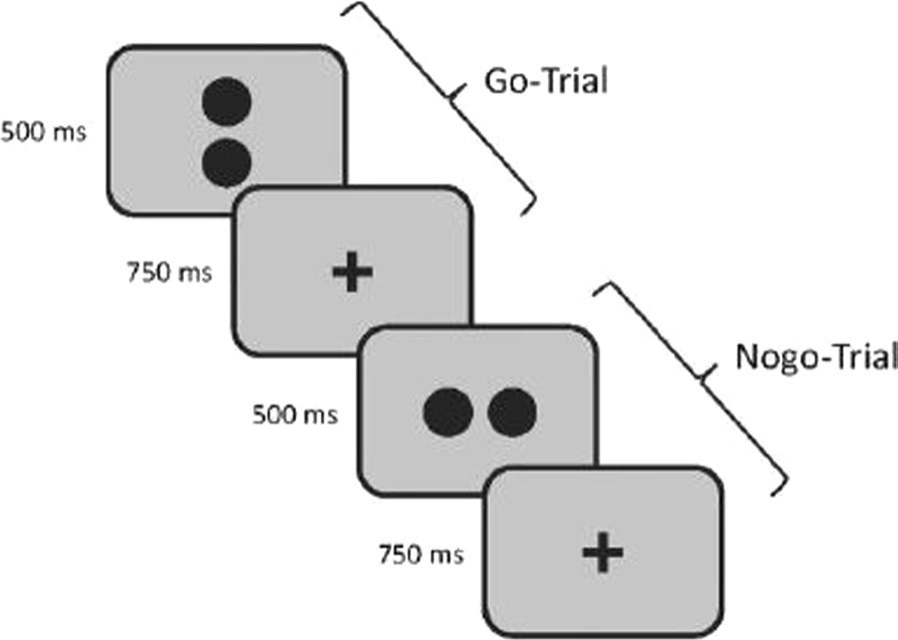
Go-nogo task

Inhibitory control is measured using a go/no-go task (Fig. 3) where participants are presented with 320 trials (280 go and 40 nogo trials) of stimuli containing two dots. Each dot pair is displayed for 500 ms and is arranged horizontally or vertically. Horizontally arranged dots indicate go-trials where participants have to press the response key as fast as possible while participants are instructed to withhold when seeing vertically arranged dots. Since there is evidence that participants balance the speed-accuracy trade-off differently [95], the dependent measure of the go/nogo task is the balanced integration score (BIS). This score is calculated in two steps. First, the responsive times (RTs) as well as the proportions of correct responses (PCs) are standardized. Second, one standardized score is subtracted from the other [96].

**Fig. 4:**
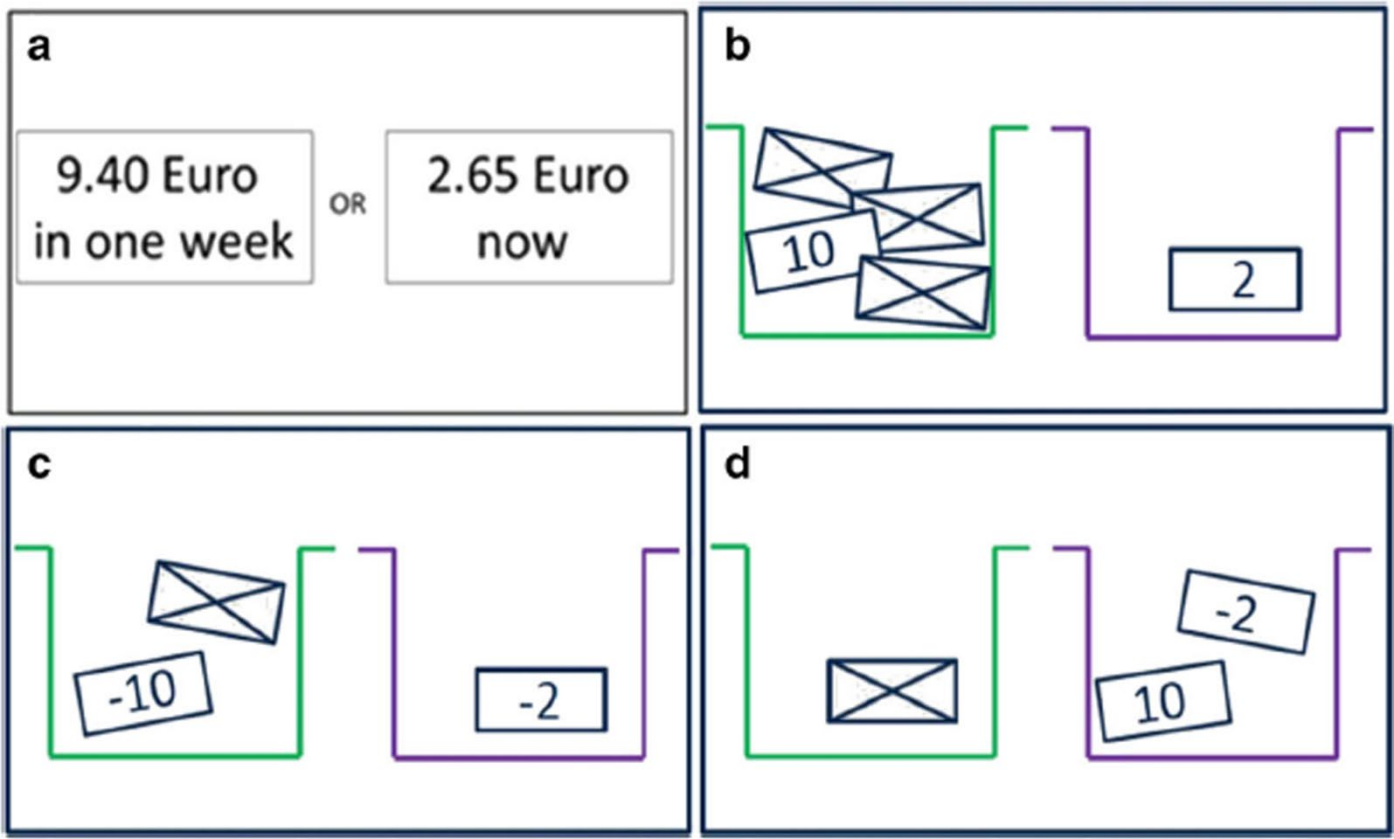
Schematic overview of the tasks in the decision-making battery. **a** Delay discounting task. **b** Probability discounting for gains. **c** Probability discounting for losses. **d** Mixed gambles task [66]

The delay discounting task as measure of impulsivity was taken from a task battery developed by Pooseh et al. ([65]; MATLAB scripts available from https://github.com/spooseh/VBDM) and is described in detail in Kräplin et al. [66]. The task consisted of 30 trials. Participants had to decide between a smaller financial gain delivered sooner and a larger financial gain delivered later. The two options were simultaneously presented on a computer screen using the Psychophysics Toolbox [97] in MATLAB R2018a (MathWorks Inc., Natick, MA). Between the shorter and later choice options, delays were 3, 7, 14, 31, 61, 180, and 365 days. Monetary gains ranged from 0.30 to 10 €. A Bayesian adaptive algorithm was implemented. This way, the parameter estimation is updated after each trial and serves as the basis for the calculation of the options in the next trial. The method was used to determine the most informative offers nearest to the individual’s point of indifference between two choice alternatives (i.e. indifference point). Thus, decision-making parameters can be efficiently inferred without the use of post-hoc parameter estimations. A hyperbolic value function was generated to describe the decline of subjective values of delayed reward according to the discounting rate k (Mazur 1987). Individuals with higher impulsivity are assumed to display higher k values (Fig. 4).

### Stress induction

Stress is induced with the Trier Social Stress Test (TSST) [98] as one of the most frequently inserted research tools for the induction of acute psychosocial stress in experimental, laboratory research worldwide. The TSST is a standardized laboratory protocol, which provides a reliable and ecologically valid stressor [98]. The TSST contains elements of social evaluative threat and uncontrollability, which are associated with high cortisol responses [99]. The test is divided in three equal five-minute parts. It begins with a preparation period, followed by a free speech for a job interview and finishes with an arithmetic task. All tasks are held in front of a two-person audience. The TSST leads to robust changes in the hypothalamus–pituitary–adrenal (HPA) axis and the autonomic stress response compared to other stress induction paradigms [39, 100].

In the control condition, subjects participate in a Placebo-TSST, which is comparable in time and task division but without any audience and stress exposure for the participants [101]. It starts with a preparation period, followed by a free speech about the last vacation and finishes with a simple task of counting forward. Furthermore, participants are standing during the two tasks. This creates a setting that is as close as possible to the TSST, but does not contain stressful components (evaluative threat and uncontrollability).

### Ad-libitum taste test

After completion of the behavioral tasks and the intervention, participants are asked to take part at an ad-libitum taste test as a covert measure for alcohol consumption. The ad-libitum taste test is a widely used method, which provides an unobtrusive and indirect measure of participants’ motivation to drink alcohol [84]. All participants are given two 0.33 l glasses of beer (two brands each containing 5% alcohol) and two 0.33 l glasses containing different soft drinks. Participants are instructed that they have 15 min to taste each glass to rate qualities about each drink (e.g. gassy, bitter). Participants are told to drink whatever amount necessary to make accurate judgements. The dependent variable is the amount of alcoholic beverage (beer) consumed and can range between 0 and 666 ml (equals 26.64 g ethanol). Non-alcoholic drinks are presented to control for the potential effect of thirst. The ad-libitum taste test is a valid method for the assessment of alcohol intake in the laboratory supported by strong associations between ad-libitum consumption and typical alcohol consumption [84]. It is also robust against several potential confounders such as time of day or participant awareness [84]. The taste test has been used to investigate a number of potential influences on alcohol consumption, including alcohol cues [102, 103, 104], impulse control [105, 106], and social influences [107], and it has been used to establish initial proof of concept for novel behavioral interventions [108, 109, 110].

### Procedure

**Fig. 5:**
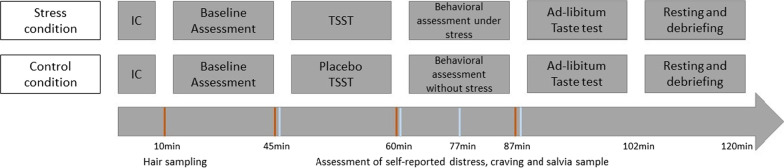
Study procedure. *Note*: *IC* Informed consent, *TSST* Trier social stress test

The main assessments are conducted between 14–20 p.m. in order to reduce the variance in biological measures (e.g. saliva cortisol) due to diurnal rhythms [111]. It is also likely that the willingness to drink alcohol is smaller in the morning than in the evening while there is no influence of day time on alcohol consumption in the ad libitum taste test between 14p.m. and 20p.m. [84]. Figure 5 gives an overview of the main study procedure. First, participants are asked to provide written informed consent. Participants’ absence from alcohol is verified by taking a breathalyzer reading with any value above zero leading to the immediate end of the examination. Hair strands for basal cortisol secretion are taken scalp-near from a posterior vertex position to be able to reflect basal cortisol secretion within two months prior to the respective assessment point. Then participants complete the baseline questionnaires including the measures of the proposed moderators (childhood maltreatment, trait impulsivity) and variables that might affect the associations of interest (usual alcohol consumption, drinking motives, stressful life events, trait anxiety, difficulties in emotion regulation, psychological flexibility). Subsequently, participants either take part in the stress induction (experimental condition) or placebo intervention (control condition) followed by behavioral assessments. Deviating from previous TSST protocols, the stress condition is maintained during the behavioral assessments. Therefore, participants are instructed that the TSST panel remain observing and evaluating the given performance during the computer tasks and further the camera is still pointed on the participant. The ad libitum taste-test is the last assessment of the procedure. After the taste test, all participants are debriefed about the true study purposes including the TSST procedure. Moreover, repeated breathalyzer readings are taken until blood alcohol concentration reaches 0.0‰ in two consecutive measures. Participants willing to leave before blood alcohol concentration reaches 0.0‰ have to confirm that they do not drive when leaving the laboratory. Participants who insist to leave with a blood alcohol concentration still being higher than 0.4‰ (only expected in rare cases) are sent home with a taxi.

### Sample storage, biochemical analyses and data preparation

The saliva samples are taken using salivette ‘code blue’ devices (Sarstedt, Nümbrecht, Germany) directly before the intervention and at three time points after the intervention (Fig. 5). Saliva samples are stored at − 20 °C in a laboratory freezer. After thawing, saliva samples will be centrifuged for 10 min at 4000 rpm. Salivary cortisol concentrations will be determined using a commercially available chemiluminescence assay (CLIA, IBL-Hamburg, Germany). Concentrations of salivary alpha-amylase will be detected by using an in-house enzyme kinetic method according to the protocol described in [63]. Hair cortisol concentrations will be determined via liquid chromatography tandem mass spectrometry (for detailed information on analysis methods see [56]).

Dimensional variables that are not normally distributed (expected e.g. for hair cortisol concentration, salivary cortisol and alpha amylase) will be Box–Cox transformed towards normal distribution. For all biological and behavioral variables, participants with outlying values of more than three standard deviations above the mean will be excluded from the respective analysis. Besides, robust linear regressions will complement conventional linear regressions because they down weight observations with large residuals to meet the assumption of equal variances of residuals. Composite measures of the entire cortisol secretion during the TSST (area under the curve with respect to ground; AUC_G_) and the cortisol stress reactivity (area under the curve with respect to increase; AUC_i_) will be calculated [112]. Analysis with these variables will be adjusted for initial cortisol concentration to alleviate confounding risk as AUC variables may be comprised of variance due to stress reactivity and stress-unrelated HPA axis activity [113]. All analysis including cortisol secretion during the TSST will be run twice with all participants in the first and with exclusion of non-responders to the TSST (increase of 1.5 nmol/l compared to baseline [114]) in the second run. With regard to alpha-amylase, both AUC measures and peak minus baseline levels will be calculated.

### Statistical analyses

Main effects of stress exposure (stress vs control group) on alcohol consumption (amount of alcoholic beverage consumed) will be determined using linear regressions adjusting for the amount of non-alcoholic beverages consumed (which reduces unspecific variance in outcome). However, in case of considerable by chance differences in baseline characteristics between the two groups despite randomization, these characteristics will be included in the regression model if they are associated with alcohol consumption. To address potential biases related to missing data, we will conduct sensitivity analyses using multiple imputation.

Moderation analyses: Moderators are defined causally [50]. Linear regressions with interaction terms will be applied to test whether stress effects on alcohol consumption are moderated by childhood maltreatment, hair cortisol concentration and trait impulsivity (with main effects terms and interaction term, e.g. group × childhood maltreatment). Significant interactions indicate that a respective factor (moderator) predicts different effects of stress on alcohol assumptions. To approach causal conclusions, we will fit these models again while adjusting for shared factors of moderators and outcomes (e.g. previous stressful events, previous alcohol use) [50].

Mediation analyses: Mediators are also defined causally according to the counterfactual definition of Robins and Greenland [115] that is implemented in the ‘paramed’ package in Stata. This module allows dividing the estimated total stress effect (stress vs. control group) on alcohol consumption into a direct effect and an indirect effect mediated through stress reactivity (saliva cortisol, alpha amylase, self-reported stress), impulsivity (delay discounting), inhibitory control, and motivational sensitization (attentional bias). Mediation analyses will be adjusted for putative sociodemographic (e.g. age) and other shared factors of a potential mediator and outcome (e.g. time of day, preference of beer) as well as for the mentioned factors for moderation. The alpha level will be specified at two-sided 0.05. If necessary, the analyses will be repeated with robust standard errors (via the sandwich estimator) and robust linear regressions [116]].

### Study progress and preliminary feasibility data

The data collection of the presented MESA study started in December 2018. From December 2018 until March 2022 N = 623 persons participated in the online screening. A total of 213 complete data sets have been collected so far. All participants are male and between 18–40 (*M* = 25) years old. 97 of the 213 participants took part in the stress condition, stratified in 40 risky drinkers and 57 regular drinkers. Further, 117 participants took part in the control condition stratified in 36 risky drinkers and 81 regular drinkers. More than half reported they were university students.

Due to the Covid-19 pandemic, the study was paused in the beginning of March 2020 in order to protect the safety and health of all personnel involved in the study and to comply with legislative regulations. The laboratories reopened in September 2021 and data collection was continued.

## Discussion

The present MESA study was developed in response to the incomplete understanding of the underlying mechanisms of the relationship between stress and alcohol consumption. As pointed out, there is a significant amount of people suffering from AUDs with tremendous consequences for the individual as well as for society and health care systems. There is need for preventive interventions at the biological, psychological or social level for individuals at high risk of problematic alcohol consumption before the manifestation of AUD. Research to date has focused primarily on secondary prevention, which aims to prevent AUD progression and relapse, and tertiary prevention, which aims to minimize functional deterioration in chronic AUDs [117]. The present study focuses on the identification of targets for primary prevention, which is focused on the protection of healthy individuals, and may be provided on a universal, selective or indicated level. The various tasks designed to examine different, potential moderators and mediators can then be used to develop interventions and provide information for the at-risk population. The identification of specific mediators is of key importance as they help to elucidate what mechanisms underlie the association between stress and alcohol consumption. Knowledge about specific mechanisms are of high relevance as it can be used to allocate existing interventions. For example, there are already trainings for many of the investigated mediators (e.g. Attentional Bias Modification, Inhibitory Control trainings), which, should these factors prove to be relevant, could then be specifically adapted and applied in the context of stress [118, 119, 120, 121]. It can also be used to develop novel interventions that might be useful to prevent stress-related alcohol consumption. Identifying specific moderators will help to tailor these preventive interventions to at high-risk individuals, which increases their potential efficacy and cost-effectiveness.

Given its focus on internal validity using a carefully controlled design in a laboratory setting, external validity will be a limitation of this study. Thus, findings will have to be complemented by investigations in real world settings to make definite conclusions about the association between stress and alcohol use and its underlying mechanisms. This could be achieved for example with ecological momentary assessments, which have shown good feasibility in a couple of promising recent studies on stress-related alcohol use and the role of craving, alterations in mood and inhibitory control [122, 123, 124, 125].

Taken together, the presented study has a high potential to advance our understanding of stress-related alcohol use. In the long-term, it could stimulate the development of tailored preventive interventions and contribute to a reduction of problematic alcohol use.

## Data Availability

The data will be made available on the OSF after completion of the first data analyses.

## Abbreviations

AUC: Area under the curve
AUC_G_: Area under the curve with respect to ground
AUC_I_: Area under the curve with respect to increase
AUD: Alcohol use disorder
BIS: Balanced integration score
DERS: Difficulties in emotion regulation scale
DMQ-R: Drinking Motive questionnaire—revised
HPA-axis: Hypothalamic–pituitary–adrenal axis
MBDF: Multidimensional Mood State Questionnaire
MESA: Underlying mechanisms in the relationship between stress and alcohol consumption in regular and risky drinkers
PCs: Proportion of correct responses
PSS: Perceived Stress Scale
RTs: Response times
SNS: Sympathetic nervous system
STAI: State-trait-anxiety-inventory
TSST: Trier Social Stress Test
